# HMGB1 mediates synaptic loss and cognitive impairment in an animal model of sepsis-associated encephalopathy

**DOI:** 10.1186/s12974-023-02756-3

**Published:** 2023-03-11

**Authors:** Xiao-Yu Yin, Xiao-Hui Tang, Shi-Xu Wang, Yong-Chang Zhao, Min Jia, Jian-Jun Yang, Mu-Huo Ji, Jin-Chun Shen

**Affiliations:** 1 Department of Anesthesiology, Jinling Hospital, Medical School of Nanjing University, Nanjing, 210002 China; 2 Department of Anesthesiology, Pain and Perioperative Medicine, The First Affiliated Hospital of Zhengzhou University, Zhengzhou, 450000 China; 3 Department of Anesthesiology, The Second Affiliated Hospital, Nanjing Medical University, Nanjing, 210011 China

**Keywords:** Sepsis, High mobility group box-1 protein, Microglia, Synaptic pruning, Cognitive impairment

## Abstract

**Background:**

Microglial activation-mediated neuroinflammation is one of the essential pathogenic mechanisms of sepsis-associated encephalopathy (SAE). Mounting evidence suggests that high mobility group box-1 protein (HMGB1) plays a pivotal role in neuroinflammation and SAE, yet the mechanism by which HMGB1 induces cognitive impairment in SAE remains unclear. Therefore, this study aimed to investigate the mechanism of HMGB1 underlying cognitive impairment in SAE.

**Methods:**

An SAE model was established by cecal ligation and puncture (CLP); animals in the sham group underwent cecum exposure alone without ligation and perforation. Mice in the inflachromene (ICM) group were continuously injected with ICM intraperitoneally at a daily dose of 10 mg/kg for 9 days starting 1 h before the CLP operation. The open field, novel object recognition, and Y maze tests were performed on days 14–18 after surgery to assess locomotor activity and cognitive function. HMGB1 secretion, the state of microglia, and neuronal activity were measured by immunofluorescence. Golgi staining was performed to detect changes in neuronal morphology and dendritic spine density. In vitro electrophysiology was performed to detect changes in long-term potentiation (LTP) in the CA1 of the hippocampus. In vivo electrophysiology was performed to detect the changes in neural oscillation of the hippocampus.

**Results:**

CLP-induced cognitive impairment was accompanied by increased HMGB1 secretion and microglial activation. The phagocytic capacity of microglia was enhanced, resulting in aberrant pruning of excitatory synapses in the hippocampus. The loss of excitatory synapses reduced neuronal activity, impaired LTP, and decreased theta oscillation in the hippocampus. Inhibiting HMGB1 secretion by ICM treatment reversed these changes.

**Conclusions:**

HMGB1 induces microglial activation, aberrant synaptic pruning, and neuron dysfunction in an animal model of SAE, leading to cognitive impairment. These results suggest that HMGB1 might be a target for SAE treatment.

## Introduction

Sepsis-associated encephalopathy (SAE) is a diffuse cerebral dysfunction caused by sepsis without direct central nervous system (CNS) infection or other types of encephalopathy [1]. SAE manifests as varying degrees of impaired consciousness, from mild delirium to coma, along with electroencephalographic (EEG) changes [2, 3], which severely reduces patients’ quality of life, increases mortality rates, and places tremendous economic pressure on society, families, and individuals [4]. Previous studies have shown that SAE development may be related to neuroinflammation, blood‒brain barrier (BBB) disruption, abnormal synaptic function, and mitochondrial dysfunction [5–7]; however, the exact mechanism remains unclear.

High mobility group box-1 protein (HMGB1) is a nonhistone DNA binding protein present in the nucleus of eukaryotic cells. HMGB1 is released passively from damaged cells and is actively extracellularly secreted by activated immune cells [8]. When immune cells are exposed to microbe-associated molecular patterns, pathogen-associated molecular patterns, and endogenous inflammatory mediators, HMGB1 activates immune cells and mediates the inflammatory response [9]. One recent study demonstrated that pregabalin ameliorates microglial activation and neuronal damage by blocking the HMGB1 signaling pathway in radiation-induced brain injury [10]. HMGB1 is also involved in cognitive impairment-related diseases, such as Alzheimer’s disease (AD) and traumatic brain injury (TBI) [11, 12]. Notably, HMGB1 is a late mediator of inflammation in sepsis [13]. In an animal model of SAE, the serum levels of HMGB1 are increased in sepsis survivors, which remains elevated for at least 4 weeks after CLP [14, 15]. Inflachromene (ICM) is a small molecule that can block cytoplasmic localization and extracellular release of HMGBs by perturbing its post-translational modification [16]. ICM treatment inhibits the cytosolic translocation of HMGB1, which is confirmed by genetic experiments based on the silencing of HMGB1 [17]. A previous study also revealed that using ICM can inhibit HMGB1 release thus limiting fructose 1,6-bisphosphatase 1-dependent hepatic stellate cell activation, the development of the secretory phenotype and tumor progression [18]. However, the specific mechanism by which HMGB1 results in cognitive impairment in SAE remains unclear.

Microglia, the primary innate immune cell population in the brain, can actively release HMGB1. HMGB1 can bind to microglial surface receptors to activate downstream inflammatory pathways and promote microglial activation [9]. Microglial activation plays essential roles in immune surveillance, synaptic plasticity regulation, and maintaining dynamic homeostasis within the CNS [19]. Microglia are activated under pathological conditions, inducing synaptic loss, neuron dysfunction, and neural circuit disruption [20, 21]. These abnormalities might contribute to the pathogenesis of cognitive impairment in many diseases. Therefore, we hypothesized that HMGB1 secretion mediates microglial activation, aberrant synaptic pruning, and neuronal dysfunction, ultimately leading to cognitive impairment in SAE mice.

## Materials and method

### Animals

C57BL/6JGpt male mice (10–12 weeks, 20–30 g) were provided by Jiangsu Jicui Pharmachem Biotechnology Co. All mice were housed under standard laboratory conditions with an automatically controlled temperature of 22 ± 2 °C, 55–65% humidity, and 12 h–12 h light–dark cycle. Four to 5 mice were housed per cage, with unlimited access to food and water.

### SAE model

SAE was induced by cecal ligation and puncture (CLP). The mice were anesthetized by intraperitoneal injection of 1% pentobarbital sodium. A 1.5 cm midline incision was performed in the abdomen to expose the cecum. The exposed cecum was ligated with 4-0 sutures below the ileocecal valve (5 mm from the cecal tip). Then, a 22 G needle was used to puncture the cecum, and a small volume of feces was gently squeezed out. After the cecum was returned to the abdominal cavity, the abdomen was closed in sequence. The mice were resuscitated immediately by hypodermic injection of normal saline (20 ml/kg), placed on a heat blanket and returned to the original cage after awakening from anesthesia. The same incision was cut in the abdomen to expose the cecum in the sham group mice, and no ligation or perforation was performed.

### Experimental design and drug treatment

**Fig. 1:**
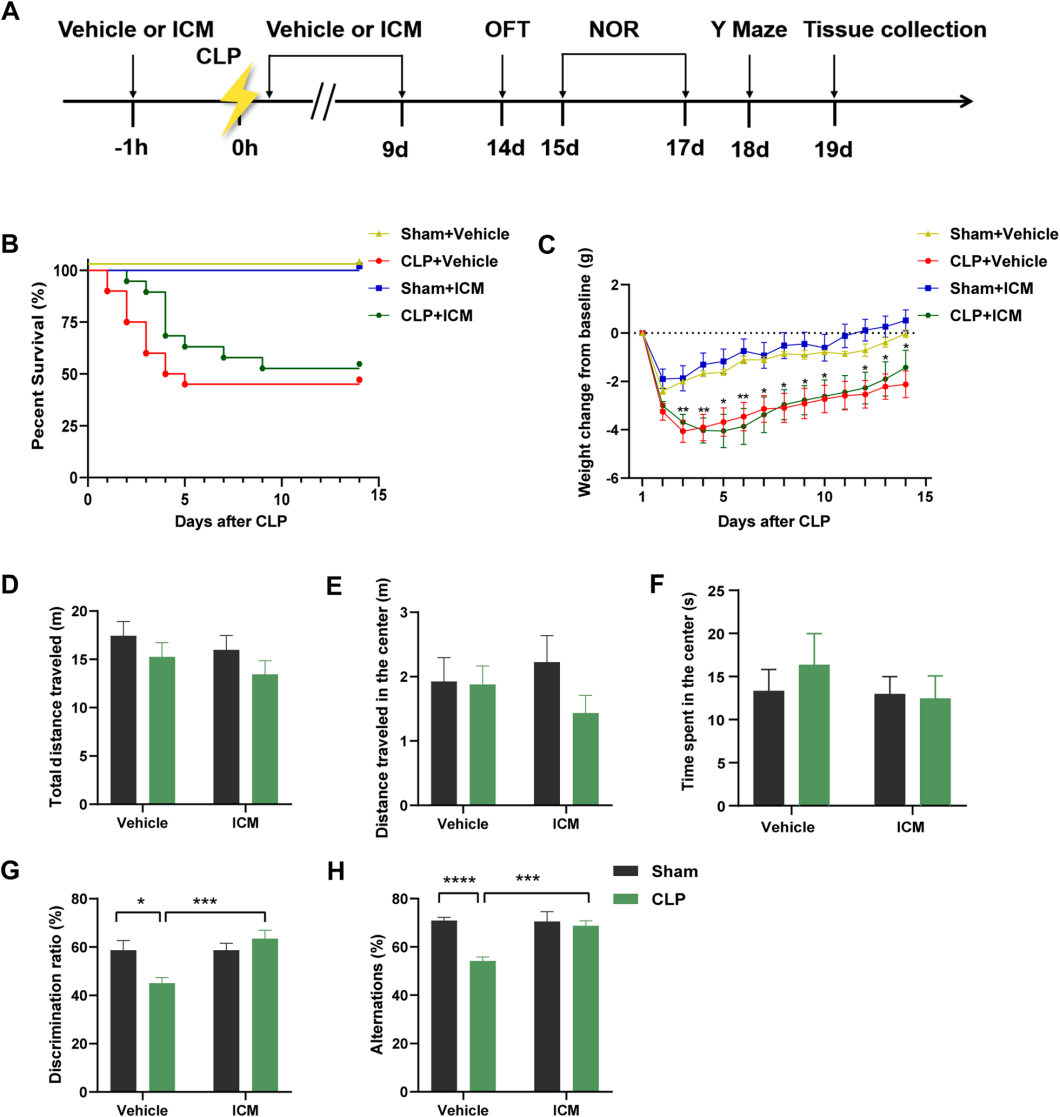
ICM ameliorated sepsis-induced cognitive impairment. **A** Flow chart of the experiment. **B** Comparison of the mouse survival rates among the four groups. **C** Comparison of the mouse body weights among the four groups. **D**–**F** OFT performance among the four groups. **G** NOR performance among the four groups. **H** Y maze test performance among the four groups. Data are presented as the mean ± SEM (n = 8–12 mice per group). *P < 0.05, **P < 0.01, ***P < 0.001, ****P < 0.0001 vs. the indicated groups

Mice were randomly assigned to the sham + vehicle group (sham + vehicle), CLP + vehicle group (CLP + vehicle), sham + inflachromene (ICM) group, or CLP + ICM group (CLP + ICM). ICM (Cayman, USA, 10 mg/kg) or vehicle (distilled water containing DMSO and PEG400) was administered intraperitoneally (*i.p.*) daily for 9 consecutive days beginning 1 h before CLP operation [22]. The experimental protocol is presented in Fig. 1A.

### Open field test

The open field test was performed to evaluate the locomotor activity of mice. A white open box (40 × 40 × 40 cm) divided into 16 squares was used. Each mouse was placed in the center of the box and allowed to explore for 5 min freely. The total distance traveled in the arena was recorded. The chamber was cleaned with 75% ethanol between each trial.

### Novel object recognition

New object recognition tests were carried out in a 40 × 40 × 40 cm white open box. The task procedure consists of the habituation, learning, and test phases. (1) In the habituation phase, mice were placed in the open box and allowed to explore freely for 10 min; (2) in the learning phase, after 1 day of habituation, mice were placed in the open box in the presence of two identical objects (A + A). (3) The test phase was conducted 24 h after the learning phase. One of the objects was replaced by a novel object, B, with a distinctively different color and shape. The length of time spent exploring familiar and novel objects was measured. The Discrimination Ratio was calculated as the new object exploration time/(new object exploration time + old object exploration time). The chamber and objects were cleaned with 75% ethanol between each trial.

### Y maze

A Y maze test was conducted to measure spatial working memory. In this experiment, a Y-maze with three arms (A, B, and C) at a 120° angle was used. Each mouse was placed into the center of the maze at the beginning of the experiment and allowed to explore freely for 8 min. Spontaneous alternation was defined as three consecutive entries into different arms, e.g., ABC, BCA, and CAB. The percentage of spontaneous alternation was used to assess working memory ability. The percentage of spontaneous alternation was calculated as spontaneous alternation number/(total number of entries-2) × 100%. Ethanol (75%) was used to clean the maze between each trial.

### Immunofluorescence

Mice were deeply anesthetized by *i.p.* injection of 1% pentobarbital sodium and then transcardially perfused with ice-cold phosphate-buffered saline (PBS) followed by 4% paraformaldehyde (PFA). The brains were removed and postfixed in 4% PFA overnight and then dehydrated in 30% sucrose for 72 h at 4 °C. Brains were then sliced into 30 μm coronal sections and mounted on slides. Slices were permeabilized and blocked with PBS containing 0.3% Triton X-100 and 10% NDS for 1 h at room temperature. Primary antibodies were incubated in PBS containing 0.3% Triton X-100 and 5% NDS overnight [rabbit anti-Iba1, 1:500 (WAKO, cat#019-19741); goat anti-Iba1, 1:1000 (Abcam, ab5076); rabbit anti-HMGB1, 1:100 (Abcam, ab18256); rat anti-CD68, 1:500 (BioLegend, cat#137001); mouse anti-vglut1, 1:500 (SYSY, 135011) and rabbit anti-c-Fos, (CST, cat#2250)]. The brain sections were then washed with PBS and treated with appropriate secondary antibodies conjugated with Alexa Fluor fluorophore in PBS containing 0.3% Triton X-100 and 5% NDS for 1 h at room temperature. The sections were rewashed with PBS and counterstained with DAPI (1:1000; Beyotime Biotechnology) for nuclear staining. Slides were coverslipped with 50% glycerin. All images were acquired with Leica TCS SP5 and Zeiss LSM880 confocal microscopes.

### Golgi staining

Golgi staining was performed per the Golgi staining kit (PK401, FD NeuroTechnologies) manufacturer’s directions. Mice were anesthetized with 1% pentobarbital sodium intraperitoneally. Whole brains were dissected, immersed in solution A + B, and stored in the dark at room temperature. After 2 weeks, the brains were transferred to solution C for 3 days. One hundred millimeter-thick coronal slices were cut at room temperature with a Vibratome (Leica VT1000S) and transferred onto 1% gelatin-coated slides for staining. After staining with solution D + E + distillation-distillationH_2_O and dehydration with different concentrations of ethanol series, the slices were cleared in xylene and coverslipped with neutral balsam mounting medium. All images were acquired with an Olympus BX53 microscope and analyzed with Fiji.

### In vivo electrophysiology

The mice were anesthetized by intraperitoneal injection of 1% pentobarbital sodium and fixed on the stereotaxic apparatus after complete anesthesia. The bregma points were exposed by cutting the scalp and adjusting the plane on the axis of the front and rear fontanel line to make it on the same level. Two points on the left side of the skull were selected for the placement of cranial nails. A 2.5 × 2.5 mm bone window was made on the right skull surface, and the dura was carefully picked out with a syringe needle. An 8-channel microfilament electrode array was fixed on a brain stereotaxic instrument and slowly placed in the CA1 region of the hippocampus (anteroposterior: − 2.2 mm, mediolateral: -1.5 mm, dorsoventral: − 1.6 mm). The electrodes were fixed with bone cement after determining successful embedding. Raw data were recorded in NOR on day 14 after surgery. In our study, the bands were classified as delta oscillations (1–4 Hz), theta oscillations (4–8 Hz), alpha oscillations (8–12 Hz), beta oscillations (12–30 Hz), and gamma oscillations (30–90 Hz). The signals were filtered with a passband of 0.3–300 Hz and further amplified and digitized at 2 kHz. All data analyses were performed by Neuroexplorer (Plexon Corporation, Dallas, TX) software.

### In vitro electrophysiology

After mice were anesthetized with isoflurane (5% air volume, 3 min), the whole brain was carefully removed and placed in 0 °C sucrose-rich artificial cerebrospinal fluid [ACSF; 126 NaCl, 2.5 KCl, 1 MgCl_2_, 1 CaCl_2_, 1.25 KH_2_PO_4_, 26 NaHCO_3_, 20 glucose (mM)]. The brain was coronally cut out according to the brain atlas in 0 °C sucrose-rich ACSF with 95% O_2_ and 5% CO_2_ oxygenation conditions (thickness 250 μm, blade speed 0.14 mm/s). The hippocampal slices were transferred into ACSF with sufficient oxygenation and incubated at room temperature for at least 45 min to ensure complete cell activity recovery. The hippocampal slices were placed on a Nikon orthomosaic microscope (FN-1 with a 10 × 40 water immersion objective stage). The stimulating electrode was placed in the Schaffer collateral (SC) to deliver the test and conditioned stimulation. The recording electrode was placed 200–300 µm from the stimulation electrode in the stratum radiatum of CA1. Forty to 50% of the maximum EPSP value was used as the baseline. After 10 min of baseline stabilization, LTP was induced with three strings of high-frequency stimulation (HFS) (50 Hz, 100 pulse), and the slope of the potential was normalized to the mean base value. The data were recorded through a SutterPatch-IPA2 amplifier, and data acquisition was performed by selecting signals in the range of 0–10 kHz and filtering them through a 5 kHz low-pass filter. Igor Pro9.0 was used to analyze the data [23].

### Statistical analysis

GraphPad Prism 8.0 (GraphPad Software) was used for statistical analyses. Data are expressed as the mean ± standard error (mean ± SEM). The normality of data distribution was assessed by the Shapiro–Wilk test. When comparing multiple groups, data were analyzed using two-factor ANOVA followed by Tukey’s post hoc test when appropriate. Changes in body weight from baseline were assessed using repeated-measures analysis of variance. Kaplan‒Meier survival curves and log-rank analyses were used to compare survival outcomes between different groups. Statistical significance was defined at *P* < 0.05.

## Results

### ICM ameliorated sepsis-induced cognitive impairment

No mice in the sham + vehicle group died 14 days after surgery, while the 14-day survival rate after CLP was only 45% (Fig. 1B). The survival rate slightly increased after ICM administration, but there was no significant difference between the two groups (Fig. 1B). Postoperative body weight significantly decreased after CLP, but there was no significant difference between the CLP and CLP + ICM groups (Fig. 1C). To assess locomotor activity and cognitive function, we performed OFT, Y maze, and NOR on days 14–18 after surgery. In the OFT, there was no significant difference in total distance traveled [Fig. 1D; interaction: CLP × ICM, F (1, 36) = 0.01335, *P* = 0.9087; CLP: F (1, 36) = 1.206, *P* = 0.8753; ICM: F (1, 36) = 2.537, *P* = 0.9391], distance traveled in the center [Fig. 1E; interaction: CLP × ICM, F (1, 36) = 1.246, *P* = 0.2717; CLP: F (1, 36) = 0.04634, *P* = 0.9996; ICM: F (1, 36) = 1.584, *P* = 0.7558], and time spent in the center [Fig. 1F; interaction: CLP × ICM, F (1, 36) = 0.3539, *P* = 0.5556; CLP: F (1, 36) = 0.5084, *P* = 0.8833; ICM: F (1, 36) = 0.1762, *P* = 0.7657] among the four groups (Fig. 1D, E). In the NOR test, the mice in the CLP + vehicle group spent less time with novel objects than those in the sham + vehicle group; this effect was reversed by ICM treatment [Fig. 1G; interaction: CLP × ICM, F (1, 33) = 8.644, *P* = 0.0060; CLP: F (1, 33) = 8.767, *P* < 0. 05; ICM: F (1, 33) = 1.981, *P* < 0.001]. In the Y maze test, compared with the sham + vehicle group, the CLP + vehicle group showed a significant decrease in spontaneous alternation, and this effect was reversed by ICM treatment [Fig. 1H; interaction: CLP × ICM, F (1, 38) = 10.61, *P* = 0.0024; CLP: F (1, 38) = 9.633, *P* < 0.0001; ICM: F (1, 38) = 16.25, *P* < 0.001]. These results indicate that inhibiting HMGB1 rescues cognitive impairment in septic mice.

### ICM ameliorated the abnormal secretion of HMGB1 in the hippocampus of SAE mice

**Fig. 2:**
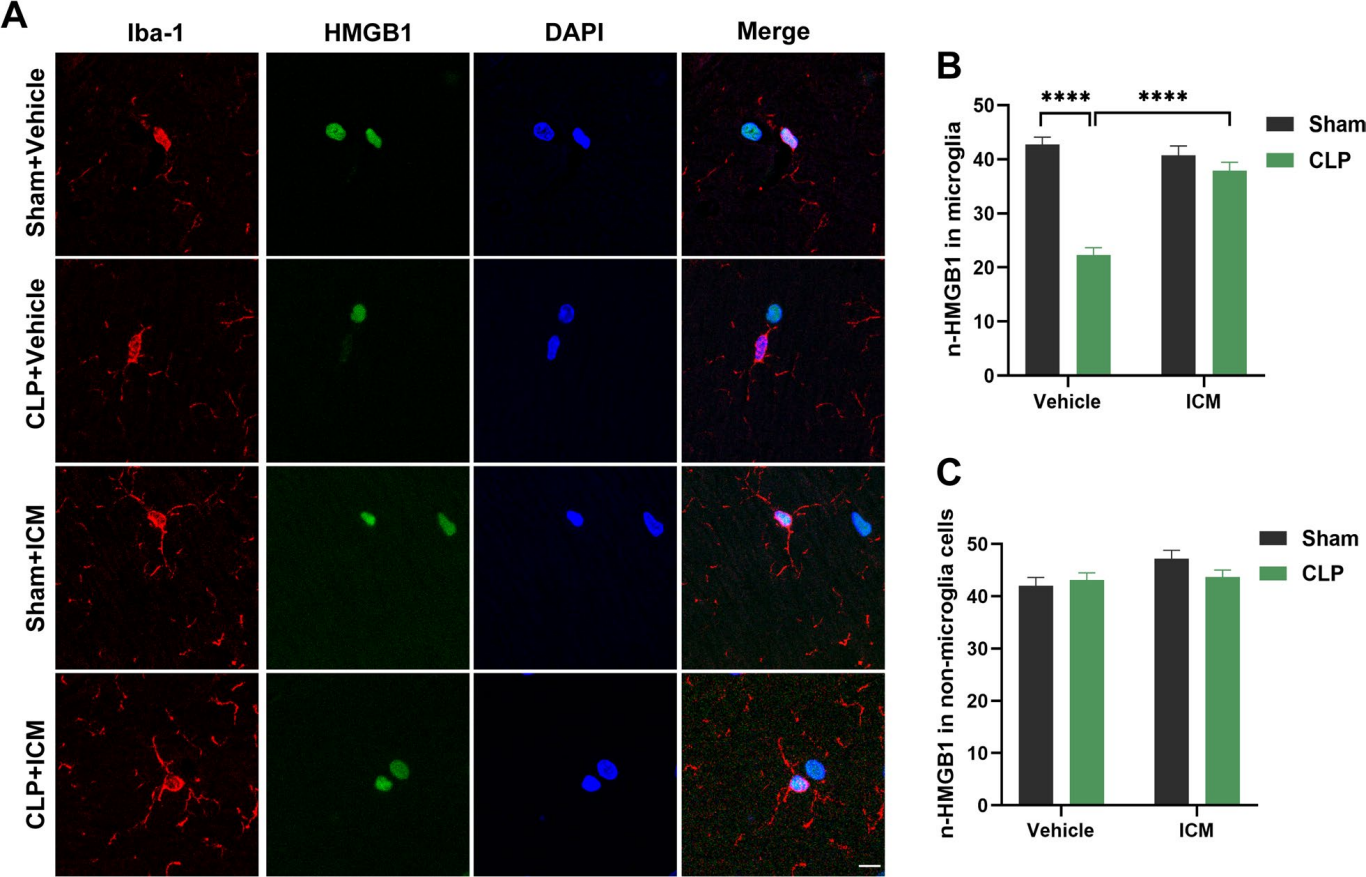
ICM ameliorated the abnormal secretion of HMGB1 in the hippocampus of SAE mice. **A** Representative images of immunofluorescence staining of Iba-1 (red), HMGB1 (green), DAPI (blue) and colocalization in the hippocampus, scale bar = 10 μm; **B**, **C** Quantification of the mean gray value of n-HMGB1 in microglia and non-microglia cells in the hippocampus among the four groups. Data are presented as the mean ± SEM (n = 3–4 mice/group). ****P < 0.0001 vs. the indicated groups

We examined the expression of nuclear HMGB1 (n-HMGB1) in the hippocampus to reveal the underlying mechanism of SAE. Compared with the sham + vehicle group, the mean gray value of n-HMGB1 in microglia was significantly decreased in the CLP + vehicle group in the hippocampus, and this effect was reversed by ICM treatment [Fig. 2A, B; interaction: CLP × ICM, F (1, 67) = 34.17, *P* < 0.0001; CLP: F (1, 67) = 20.24, *P* < 0. 0001; ICM: F (1, 67) = 59.74, *P* < 0.0001]. However, there were no significant differences in n-HMGB1 expression levels in non-microglia cells among the four groups [Fig. 2C; interaction: CLP × ICM, F (1, 68) = 2.552, *P* = 0.1148; CLP: F (1, 68) = 3.991, *P* = 0.9504; ICM: F (1, 68) = 0.7091, *P* = 0.9920]. These results suggest that HMGB1 secreted from microglia increases after CLP.

### ICM restored the changes in the number and morphology of microglia in the hippocampus of SAE mice

**Fig. 3:**
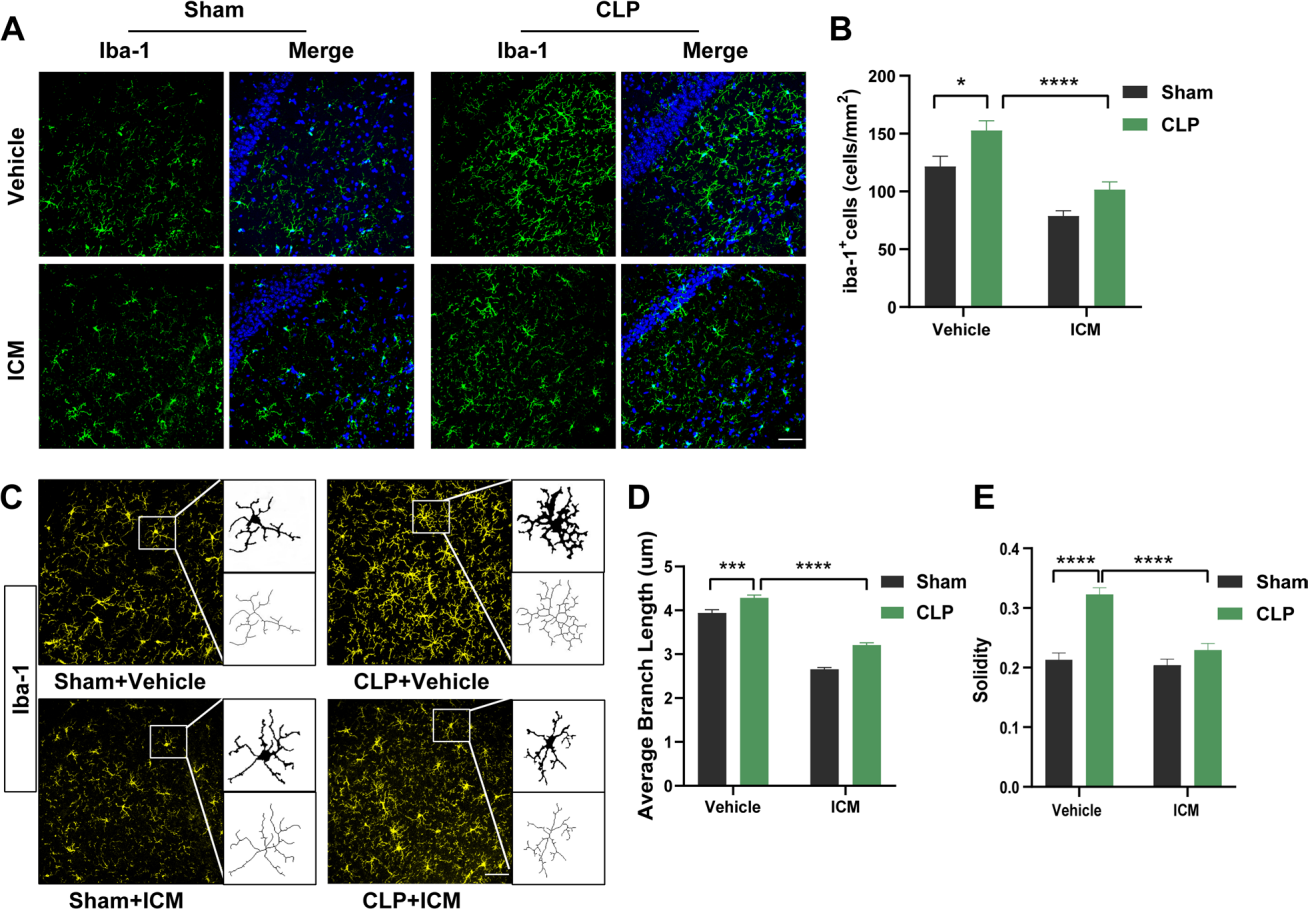
ICM restored the changes in the number and morphology of microglia in the hippocampus of SAE mice. **A** Representative images of immunofluorescence staining of Iba-1 (green) and DAPI (blue) in the hippocampus, scale bar = 50 μm; **B** quantification of the number of Iba-1^+^ cells (microglia) in the hippocampus among the four groups; **C** representative images of immunofluorescence staining of Iba-1 (yellow) and skeletonization of Iba-1^+^ cells (microglia) in the hippocampus, scale bar = 50 μm; **D** quantification of the average branch length of Iba-1^+^ cells (microglia) in the hippocampus among the four groups; **E** quantification of the solidity of Iba-1^+^ cells (microglia) in the hippocampus among the four groups. Data are presented as the mean ± SEM (n = 3–5 mice/group). *P < 0.05, ***P < 0.001, ****P < 0.0001 vs. the indicated groups

The number of microglia was significantly increased in the CLP + vehicle group compared with the sham + vehicle group, and ICM treatment reversed this effect [Fig. 3A, B; interaction: CLP × ICM, F (1, 60) = 0.3246, *P* = 0.5710; CLP: F (1, 60) = 41.44, *P* < 0.05; ICM: F (1, 60) = 13.71, *P* < 0.0001]. The microglial morphological analysis results showed that the average branch length and solidity of the microglia were significantly increased in the CLP + vehicle group compared to the sham + vehicle group; ICM treatment reversed this effect [Fig. 3C–E; D: interaction: CLP × ICM, F (1, 116) = 9.551, *P* = 0.0025; CLP: F (1, 116) = 14.96, *P* < 0.001; ICM: F (1, 116) = 10.36, *P* < 0.0001; E: interaction: CLP × ICM, F (1, 68) = 19.25, *P* < 0.0001; CLP: F (1, 68) = 28.26, *P* < 0.0001; ICM: F (1, 68) = 49.09, *P* < 0.0001]. The immunofluorescence results indicate that ICM reverses the microglial activation induced by CLP.

### ICM reversed the enhanced phagocytic activity of microglia in the hippocampus of SAE mice

**Fig. 4:**
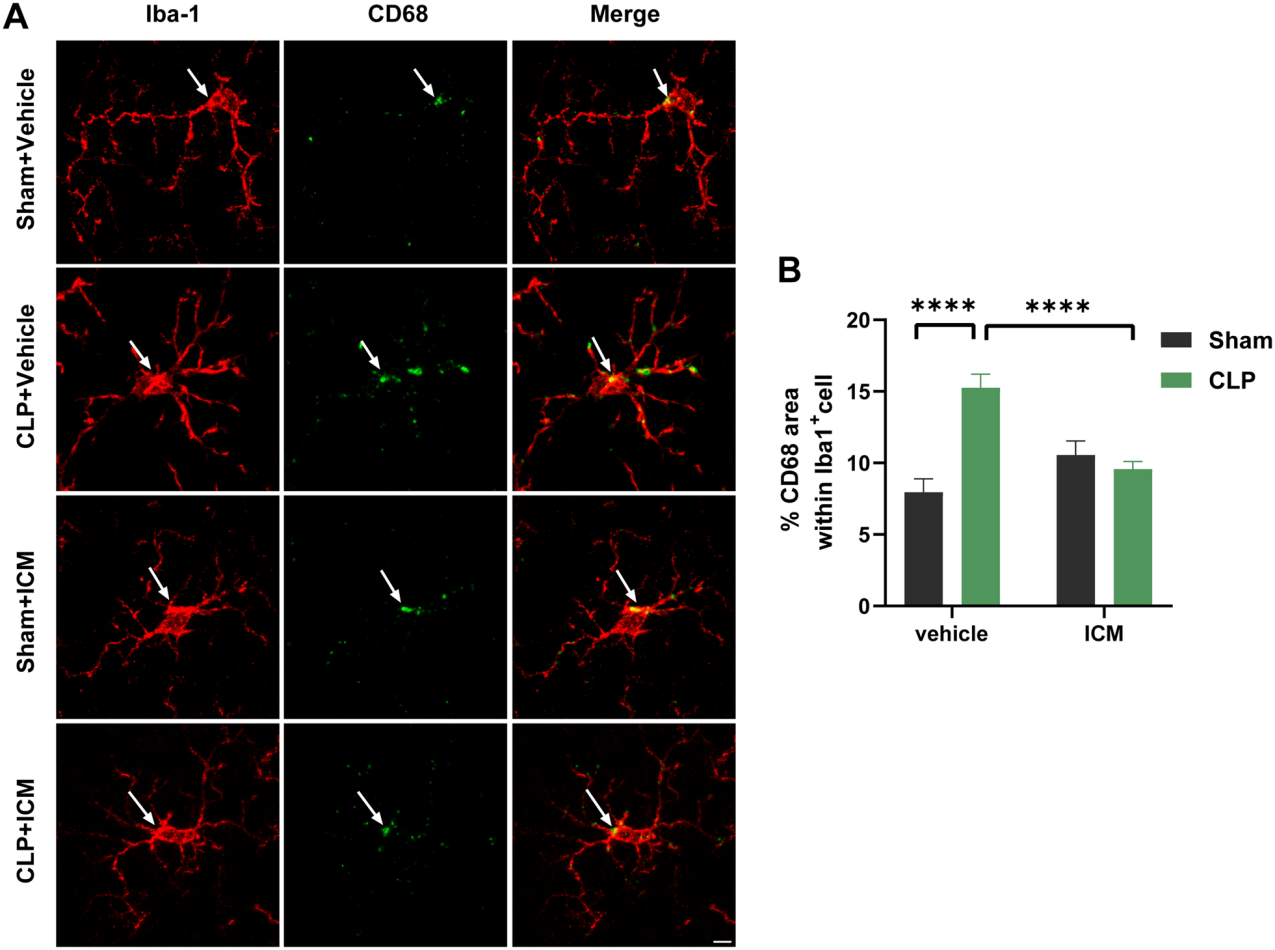
ICM reversed the enhanced phagocytic activity of microglia in the hippocampus of SAE mice. **A** Representative images of immunofluorescence staining of CD68 (green) and Iba-1 (red) and colocalization in the hippocampus, scale bar = 5 μm; **B** quantification of the percentage of CD68 area within Iba-1^+^ cells (microglia) in the hippocampus among the four groups. Data are presented as the mean ± SEM (n = 4–6 mice/group). ****P < 0.0001 vs. the indicated groups

We measured microglial CD68 levels by immunofluorescence to further assess the changes in the phagocytic activity of microglia. The area percentage of CD68 expression in Iba1^+^ cells in the CLP + vehicle group was significantly increased compared with that in the sham + vehicle group, and this effect was reversed by ICM treatment [Fig. 4A, B; interaction: CLP × ICM, F (1, 184) = 22.61, *P* < 0.0001; CLP: F (1, 184) = 3.115, *P* < 0.0001; ICM: F (1, 184) = 13.04, *P* < 0.0001]. These data suggest that HMGB1 significantly increases microglial CD68 expression and provide evidence that HMGB1 may affect microglial phagocytic activity in SAE.

### ICM reduced microglia-mediated synapse elimination in the hippocampus of SAE mice

**Fig. 5:**
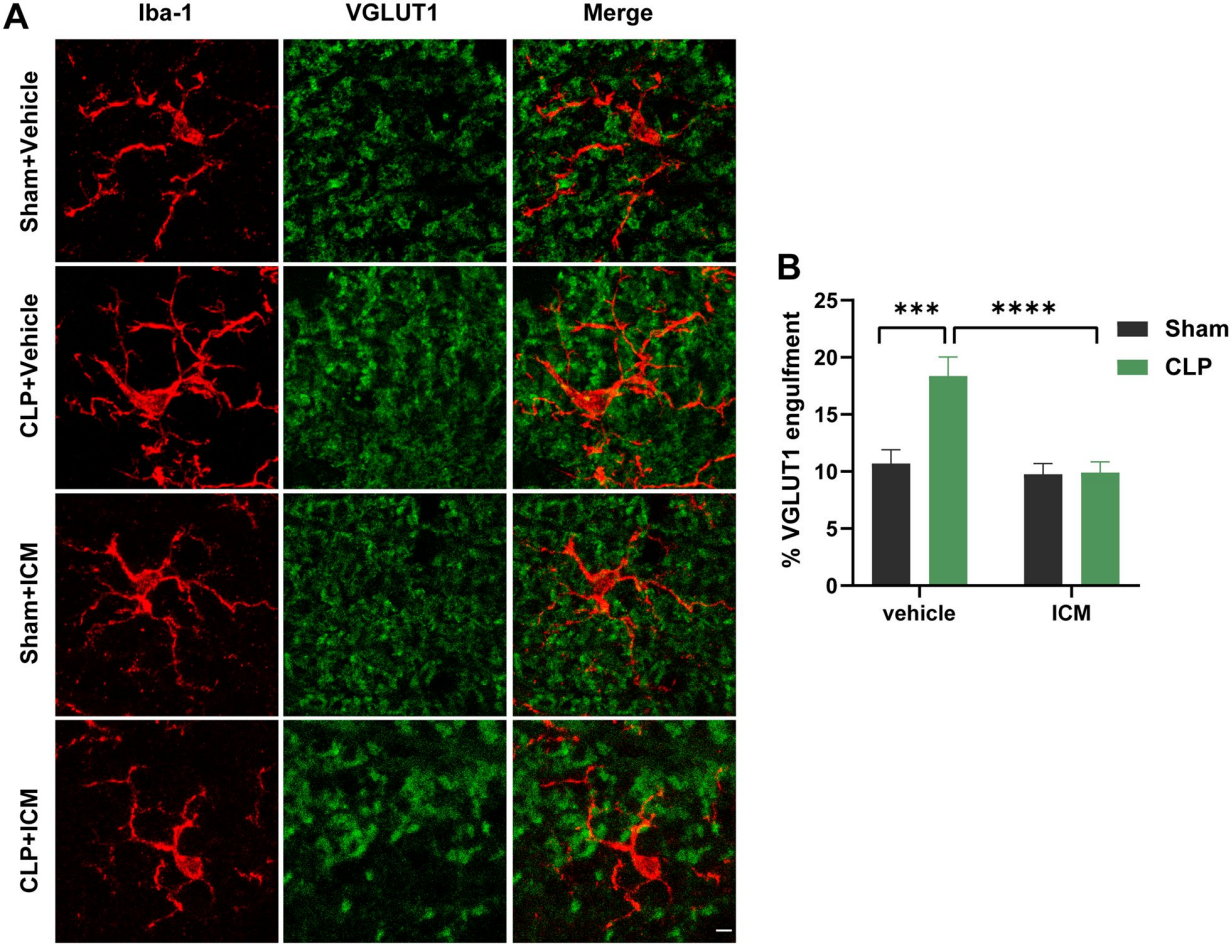
ICM reduced microglial-mediated synapse elimination in the hippocampus of SAE mice. **A** Representative images of immunofluorescence staining of VGLUT1 (green), Iba-1 (red) and colocalization in the hippocampus, scale bar = 5 μm; **B** quantification of the percentage of VGLUT1 engulfment within Iba-1^+^ cells (microglia) in the hippocampus among the four groups. Data are presented as the mean ± SEM (n = 3–5 mice/group). ***P < 0.001 and ****P < 0.0001 vs. the indicated groups

We evaluated the area of engulfed VGLUT1^+^ puncta within Iba1^+^ cells by immunofluorescence to investigate whether HMGB1-induced microglial activation leads to synapse engulfment in SAE. The results showed that Iba1^+^ cells from the hippocampus of the CLP + vehicle group engulfed significantly more VGLUT1^+^ puncta than hippocampal Iba1^+^ cells from the sham + vehicle group, and this effect was reversed by ICM treatment [Fig. 5A, B; interaction: CLP × ICM, F (1, 116) = 9.551, *P* = 0.0025; CLP: F (1, 116) = 14.96, *P* < 0.001; ICM: F (1, 116) = 10.36, *P* < 0.0001]. These data suggest that HMGB1 leads to aberrant pruning of hippocampal excitatory synapses through microglial activation.

### ICM ameliorated abnormal neuronal morphology and loss of dendritic spines in the hippocampus of SAE mice

**Fig. 6:**
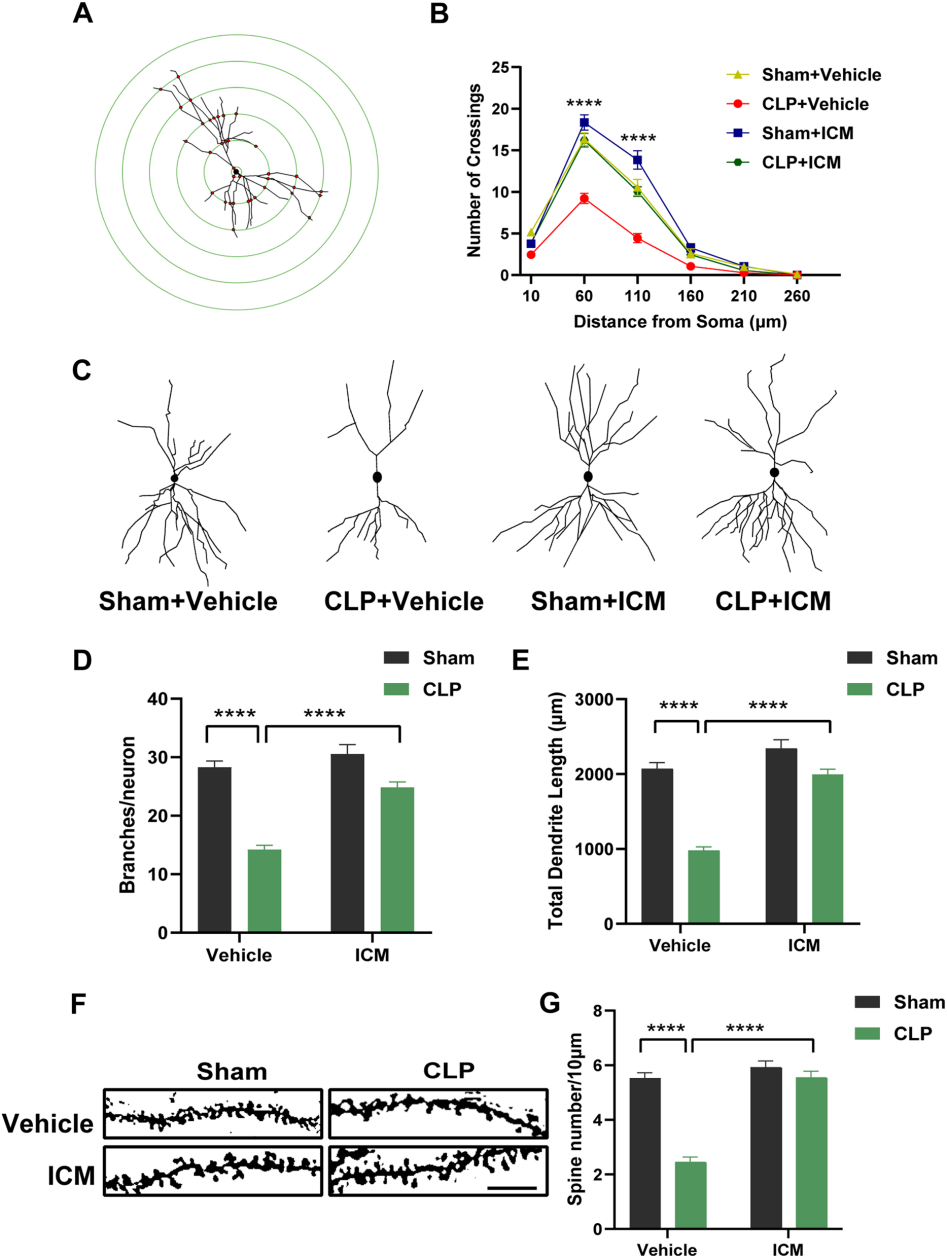
ICM ameliorated abnormal neuronal morphology and loss of dendritic spines in the hippocampus of SAE mice. **A** Sholl analysis pattern map of neuronal morphology; **B** quantification of dendritic intersections of neuronal dendrites in the hippocampus among the four groups; **C** representative images of hippocampal neuronal tracings; **D** quantification of the number of neuronal branches in the hippocampus among the four groups; **E** quantification of the total length of neuronal branches in the hippocampus among the four groups; **F** representative images of the dendritic spines of hippocampal neurons; **G** quantification of dendritic spine density of neurons in the hippocampus among the four groups, scale bar = 10 μm. Data are presented as the mean ± SEM (n = 4 mice/group). ****P < 0.0001 vs. the indicated groups

We next analyzed the morphology of Golgi-stained neurons and found that after CLP, the number of dendritic crossings of pyramidal neurons at 50 μm and 100 μm from the cell body was significantly reduced; ICM treatment reversed this effect [Fig. 6A, B; 50 μm: interaction: CLP × ICM, F (1, 68) = 10.06, *P* = 0.0023; CLP: F (1, 68) = 32.60, *P* < 0.0001; ICM: F (1, 68) = 34.23, *P* < 0.0001; 100 μm: interaction: CLP × ICM, F (1, 68) = 1.950, *P* = 0.1671; CLP: F (1, 68) = 28.30,* P* < 0.0001; ICM: F (1, 68) = 35.03,* P* < 0.0001]. Compared with the sham + vehicle group, the number of neuronal branches in the CA1 was significantly reduced in the CLP + vehicle group, and this effect was reversed by ICM treatment [Fig. 6C, D; interaction: CLP × ICM, F (1, 68) = 13.38, *P* = 0.0005; CLP: F (1, 68) = 32.02, *P* < 0.0001; ICM: F (1, 68) = 75.39, *P* < 0.0001]. Compared with the sham + vehicle group, the total length of neuronal branches in the CA1 in the CLP + vehicle group was significantly reduced, and ICM treatment reversed this effect [Fig. 6E; interaction: CLP × ICM, F (1, 68) = 20.44, *P* < 0.0001; CLP: F (1, 68) = 60.92, *P* < 0.0001; ICM: F (1, 68) = 76.45, *P* < 0.0001]. An analysis of dendritic spine density in the CA1 by Golgi staining revealed that the dendritic spine density of pyramidal neurons in the CA1 of the hippocampus was significantly reduced in the CLP + vehicle group compared with the sham + vehicle group, and ICM treatment reversed this effect [Fig. 6F, G; interaction: CLP × ICM, F (1, 68) = 42.89, *P* < 0.0001; CLP: F (1, 68) = 72.26, *P* < 0.0001; ICM: F (1, 68) = 69.57, *P* < 0.0001]. These findings suggest that HMGB1 mediates neuronal morphology damage and loss of dendritic spines.

### ICM reversed the decreased theta oscillation in the hippocampus of SAE mice

**Fig. 7:**
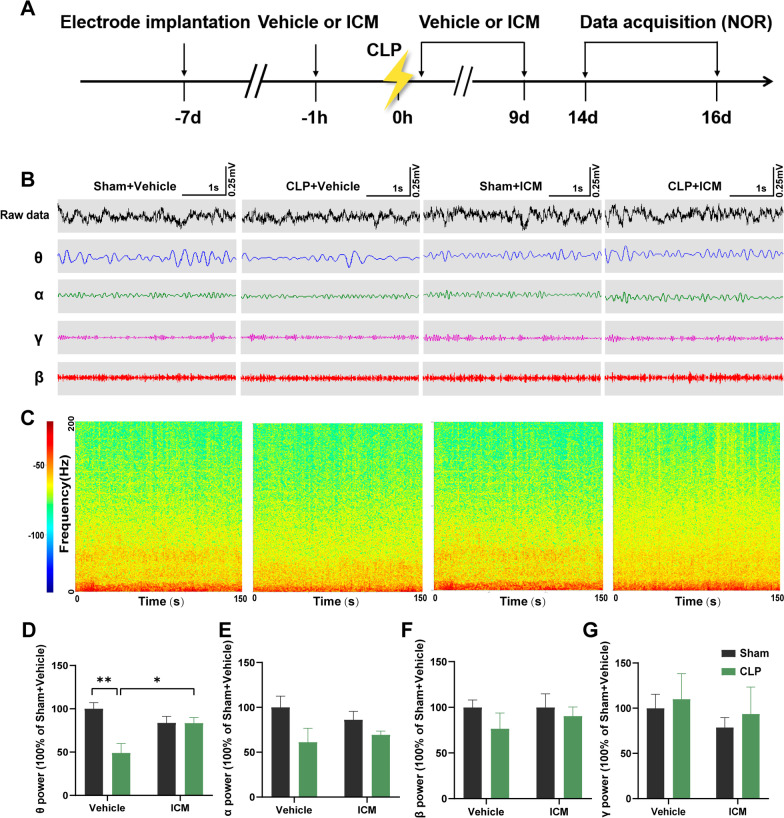
ICM reversed the abnormal theta oscillation in the hippocampus of SAE mice. **A** Flow chart of the electrophysiological experiment. **B** Representative images of local field potential and filtered theta, alpha, beta, and gamma oscillations in the hippocampus among the four groups. **C** Example power spectra of local field potential in the hippocampus. **D**–**G** Quantification of average theta, alpha, beta, and gamma oscillation power in the hippocampus among the four groups. Data are shown as the mean ± SEM (n = 3–5 mice/group), *P < 0.05 and **P < 0.01 vs. the indicated groups

We further evaluated the changes in neural oscillations in the hippocampus of SAE mice while the mice performed NOR. In the present study, the power of theta oscillation in the CLP + vehicle group was significantly decreased compared with that in the sham + vehicle group, and this effect was reversed by ICM treatment [Fig. 7D; interaction: CLP × ICM, F (1, 20) = 9.503, *P* = 0.0059; CLP: F (1, 20) = 1.198, *P* < 0.01; ICM: F (1, 20) = 9.623, *P* < 0.05]. In addition, there were no significant differences in alpha, beta, and gamma oscillation power among the four groups [Fig. 7E; interaction: CLP × ICM, F (1, 20) = 0.9751, *P* = 0.3352; CLP: F (1, 20) = 0.06195, *P* = 0.1000; ICM: F (1, 20) = 6.120, *P* = 0.9527; Fig. 7F; interaction: CLP × ICM, F (1, 20) = 0.2811, *P* = 0.6018; CLP: F (1, 20) = 0.2811, *P* = 0.5929; ICM: F (1, 20) = 1.594, *P* = 0.8757; Fig. 7G; interaction: CLP × ICM, F (1, 20) = 0.01186, *P* = 0.9144; CLP: F (1, 20) = 0.2811, *P* = 0.9894; ICM: F (1, 20) = 1.594, *P* = 0.9556]. These results suggest that inhibiting HMGB1 reverses abnormal theta oscillation in the CA1 of the hippocampus after CLP.

### ICM reversed the impairment of neuronal activity and synaptic dysfunction in the hippocampus of SAE mice

**Fig. 8:**
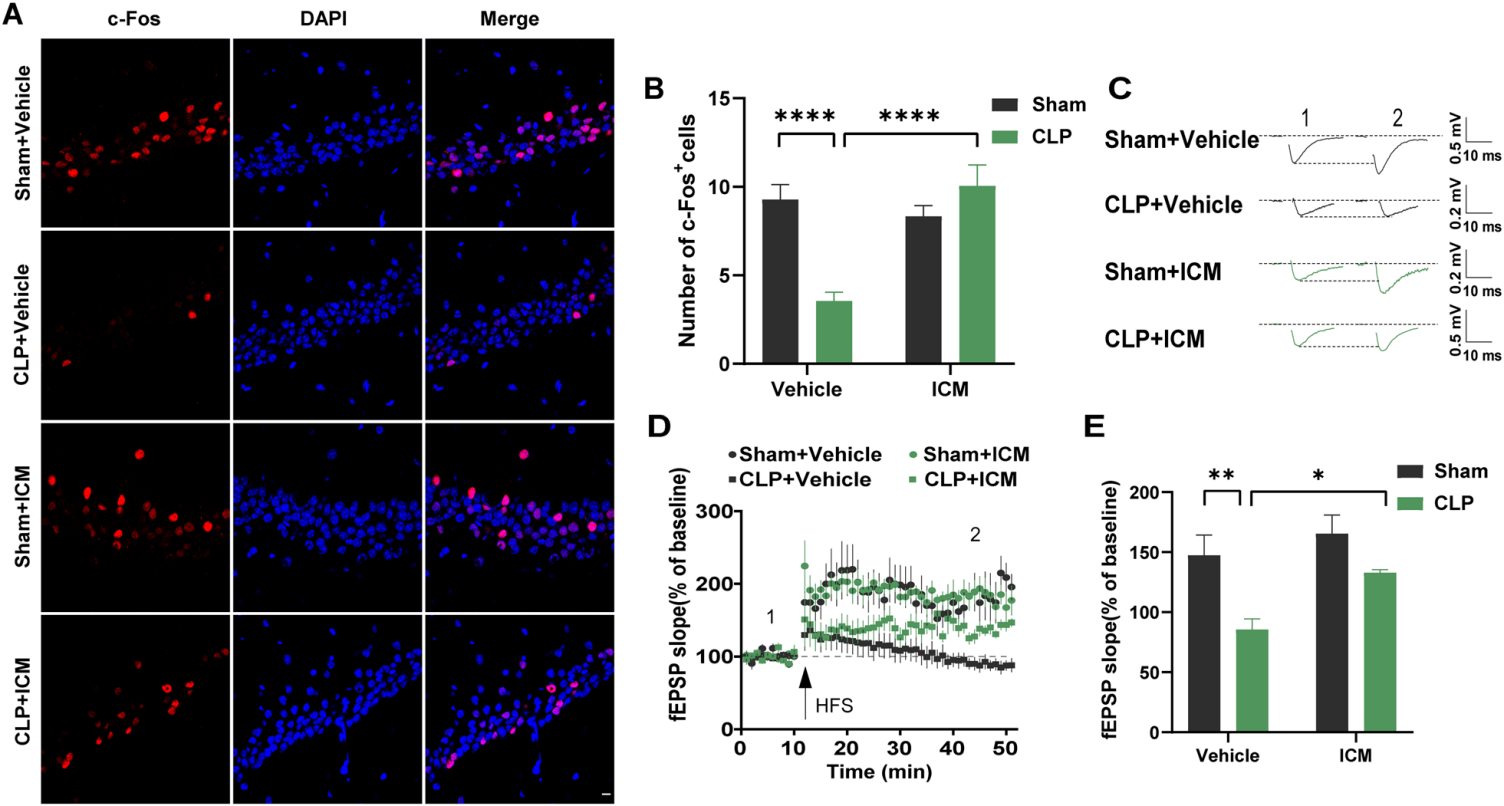
ICM reversed the impairment of neuronal activity and synaptic dysfunction in the hippocampus of SAE mice. **A** Representative images of immunofluorescence staining of c-Fos (red), DAPI (blue) and colocalization in the hippocampus, scale bar = 10 μm; **B** quantification of the number of c-Fos^+^ cells in the hippocampus among the four groups. (n = 3 mice/group). **C** Schematic representation of fEPSPs before (1) and after (2) HFS among the four groups. Horizontal calibration bars, 10 ms; vertical bars, 0.5 mV. **D** Time-dependent changes in the slope of fEPSPs before (1) and after (2) HFS in hippocampal slices among the four groups. **E** Quantification of fEPSP slopes evoked by HFS among the four groups. (n = 5–6 slices/group). Data are presented as the mean ± SEM. *P < 0.05, **P < 0.01, ****P < 0.0001 vs. the indicated groups

Given that neural oscillations are driven by fluctuations in the excitability of populations of neurons, we further examined neuronal activity in the hippocampus of SAE mice. C-Fos protein is the product of the immediate early gene that is a marker of neuronal activation (Morgan and Curran 1986; Dragunow and Faull 1989). The number of c-Fos+ cells in the CLP + vehicle group was significantly decreased compared with that in the sham + vehicle group, and ICM treatment reversed this effect [Fig. 8A, B; interaction: CLP × ICM, F (1, 68) = 20.56, *P* < 0.0001; CLP: F (1, 68) = 11.45, *P* < 0.0001; ICM: F (1, 68) = 5.935, *P* < 0.0001]. Meanwhile, HFS-induced SC-CA1 LTP was significantly reduced in the hippocampus of the CLP + vehicle group compared with the sham + vehicle group, and this effect was also reversed by ICM treatment [Fig. 8C–E; interaction: CLP × ICM, F (1, 17) = 1.560, *P* = 0.2287; CLP: F (1, 17) = 7.818, *P* < 0.01; ICM: F (1, 17) = 16.06, *P* < 0.05]. These results suggest that HMGB1 impairs neuronal activity and synaptic function after CLP.

## Discussion

**Fig. 9:**
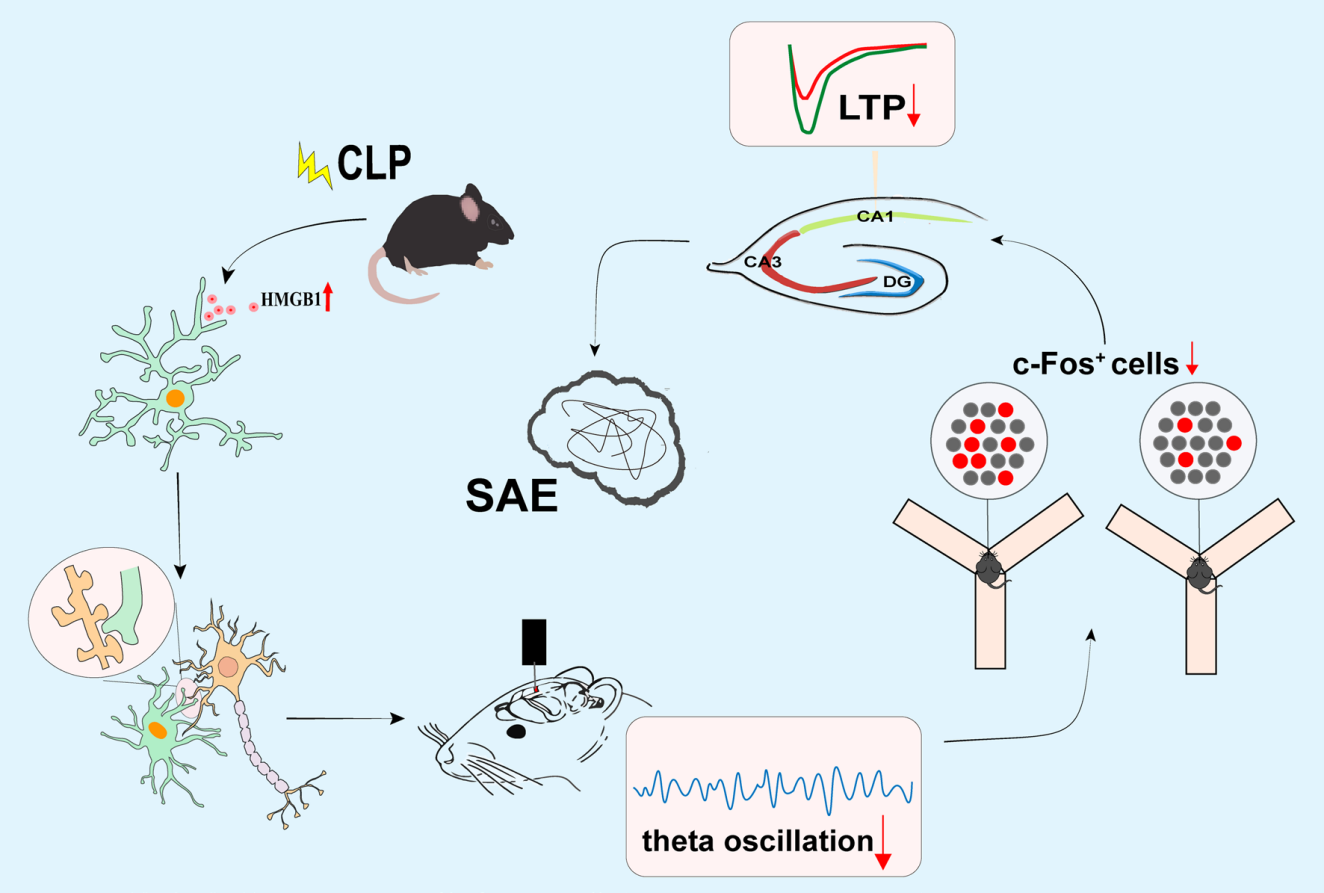
The schematic diagram illustrates that CLP induces increased secretion of HMGB1 and microglial activation, thus causing abnormal synaptic elimination and decreased theta oscillation. Abnormal theta oscillation reduces c-Fos^+^ cells and impairs LTP, ultimately leading to cognitive impairment in SAE mice

In the present study, we showed that in SAE mice, HMGB1 mediates microglial activation and induces excessive phagocytosis of synapses and neuron dysfunction, ultimately leading to cognitive impairment. Notably, inhibiting HMGB1 secretion reverses these aberrant changes and ameliorates cognitive impairment in SAE mice (Fig. 9).

SAE is a severe CNS complication of sepsis, manifesting clinically as diffuse brain dysfunction, with varying degrees of neurological symptoms ranging from lethargy to coma, and over 80% of patients demonstrate EEG abnormalities [1, 24, 25]. SAE is the most common cause of encephalopathy in medical and surgical intensive care [26], and approximately half (46%) of patients with sepsis have SAE [27]. After CLP, the surviving mice in our study exhibited impaired learning and memory, as assessed by the NOR and Y maze tests. Consistent with our results, it has been suggested that sepsis induced by CLP can lead to cognitive impairment [28]. Lipopolysaccharide (LPS) injection can also cause short- and long-term cognitive impairments through a mechanism that releases various inflammatory cytokines in the brain [29]. However, the CLP model induces a slower but more stable increase in plasma cytokines that resembles human sepsis more closely than endotoxin administration. The CLP model can also cause more prolonged and consistent cognitive impairment after sepsis relative to LPS administration. Therefore, CLP is considered the gold standard model for SAE [30].

The underlying pathological mechanisms of SAE are multifactorial and highly complex. BBB damage, cerebral microcirculation dysfunction, abnormal inflammatory response, and abnormal brain metabolism may all be involved in SAE development, but the specific mechanisms are not yet known [1]. Accumulating evidence suggests that neuroinflammation triggered by abnormal inflammatory cytokine expression plays a pivotal role in SAE. It has been reported that activation of the NLRP3 inflammasome via mitophagy inhibition in cerebral microvascular endothelial cells may promote the secretion of IL-1β into the CNS and induce neuroinflammation, leading to SAE [5]. In addition, a previous study reported that double-stranded RNA dependent protein kinase could physically interact with inflammasome components and mediates inflammasome activation, thus regulating release of HMGB1 [31]. Interestingly, HMGB1 is also a late mediator of inflammation in sepsis [13] and participates in the amplification of neuroinflammation [32]. However, there are few studies on the role of HMGB1 in SAE. HMGB1 translocates from the nucleus into the cytoplasm and is eventually released into the extracellular space. The nucleus–cytoplasm transfer of HMGB1 is a critical step in the active release of HMGB1, decreasing n-HMGB1 levels [33, 34]. In our study, we demonstrated that the expression of n-HMGB1 was significantly decreased in the hippocampus after CLP, while ICM treatment inhibited HMGB1 secretion and reduced cognitive impairment. These results indicate that inhibiting HMGB1 secretion may be a critical potential mechanism for treating SAE. Consistent with our data, HMGB1 release induces cognitive deficits in diabetes-related dementia and TBI [12, 35]. In an animal model of AD, HMGB1 induced cognitive impairment through the Sirtuin 3/superoxide dismutase 2 signaling pathway [11]. However, the mechanism by which HMGB1 induces cognitive impairment in SAE remains unclear.

Microglia are the primary immune cells in the brain and are the main source of cytokines in the CNS. Microglial activation induces a change from a resting state to a highly branched state (hyperramification), an increase in the average length of the branches and an amoebic state [36]. Our study showed that CLP increased the number and average length of microglial branches with a concomitant increased expression of CD68 in the hippocampus. These results suggest that the phagocytic ability of activated microglia is enhanced in SAE mice. Inhibiting HMGB1 secretion through ICM treatment reversed the above anomalies, suggesting that HMGB1 is a crucial factor in mediating microglial activation in SAE. Numerous studies have demonstrated that microglial activation is closely associated with cognitive impairment in AD, vascular dementia, postoperative cognitive impairment, cerebral ischemia-percussion injury, and TBI [37–40]. Additionally, under pathological conditions, the enhanced phagocytic ability of long-term activated microglia can lead to excess inflammatory mediator expression, aberrant synaptic pruning, neuronal dysfunction, or even death [41]. In our study, excitatory synapses were engulfed by microglia following CLP, and this effect was rescued by ICM administration. These findings indicate that HMGB1 causes cognitive impairment by enhancing microglial phagocytic ability and inducing excitatory synaptic engulfment. However, a previous study suggested that GABA-receptive microglia selectively prune inhibitory synapses in postnatal development and impair this microglial response, leading to behavioral abnormalities such as attention-deficit/hyperactivity disorder [42]. The reasons for this discrepancy are unknown but may be due to the different animal models used.

In the CNS, neurons can process thousands of different synaptic inputs, with dendrites playing an essential role in the brain’s neural network. Dendrites can receive input from other neurons, integrating and transmitting signals to the cell body, triggering the generation and propagation of neuronal action potentials, thus transferring information and performing complex cognitive tasks [43]. Dendritic spines are spiny protrusions of dendrites and are the primary site of synaptic connections between neurons [44]. In this experiment, we examined changes in neuronal morphology and dendritic spine density of pyramidal neurons in the hippocampus by Golgi staining. We found that the neuronal morphology was impaired, and the dendritic spine density was significantly reduced after the CLP operation, suggesting that the synaptic structural integrity of the hippocampus in SAE mice was impaired. Inhibiting the secretion of HMGB1 improved the abnormal morphological changes in pyramidal neurons and the loss of dendritic spines in the hippocampus of SAE mice. Conversely, HMGB1 can promote neurite outgrowth and cell migration during early brain development and is essential for processes such as forebrain development [45]. This discrepancy is most likely due to the the different redox state of HMGB1 which resulting in activation of different HMGB1 receptors and subsequent biological outcome [46]. The release of all-thiol (all-reduced) HMGB1 potentiates chemotaxis and promotes neurite outgrowth via binding to receptor for advanced glycation end products. During inflammation, the disulfide form of HGMB1 releasing from activated macrophage binds to Toll-like receptor 4 to induce cytokine production [46–48]. In addition, a disintegrin and metalloproteinase domain 10 is involved in dendritic spine shaping in an animal model of AD, which alters dendritic morphology, induces dendritic spine loss, and impairs information transfer between neurons [49]. Dendritic spines are essential for receiving neurotransmitters and regulating synaptic plasticity, which is the basis of learning and memory [50]. Activated microglia cause dendritic spine loss and neuronal damage or death by increasing synaptic phagocytosis, which can cause various neuropsychiatric disorders [51–53]. These results suggest a complex interaction between microglia and neurons under different conditions, promoting the formation of neuronal connections and maintaining an optimal neural network.

Neural oscillations are the rhythms of neural activity observed at different temporal and spatial scales, which are classified as delta oscillations (1–4 Hz), theta oscillations (4–8 Hz), alpha oscillations (8–12 Hz), beta oscillations (12–30 Hz) and gamma oscillations (30–90 Hz) [54]. Numerous studies have shown that neural oscillations play vital roles in perceptual, cognitive, motor, and emotional processes [55]. For example, in animal models of schizophrenia, diminished gamma oscillations have been shown to lead to impaired cognitive function [56, 57]. We detected neural oscillations of the hippocampus in mice performing NOR to further investigate the neural mechanisms underlying cognitive impairment in SAE. Interestingly, our study indicated that theta oscillation power was significantly decreased after CLP, and this effect was reversed by ICM treatment. In an animal model of AD, inhibitory and rhythmic septohippocampal activity are selectively reduced, and hippocampal theta oscillation power is impaired during a cognitive task, leading to behavioral disorders in information processing [58]. In addition, several intracranial studies have suggested that increased theta power is associated with successful encoding or retrieval [59, 60]. A previous study demonstrated that the reduction in LFP cross-frequency coupling between theta and gamma power in the hippocampus is associated with impaired LTP in an animal model of transient global cerebral ischemia [61]. Consistently, our study indicated that decreased theta oscillation was accompanied by reduced activity of neurons and impaired LTP in the hippocampus of SAE mice. These results suggest that HMGB1 mediates theta oscillation disruption and network dysregulation, thus leading to cognitive deficits in SAE.

## Conclusions

Our study demonstrated that increased HMGB1 secretion induces microglial activation, which in turn induces abnormal synaptic elimination and neuronal dysfunction in the hippocampus, ultimately leading to cognitive impairments in SAE mice. Administration of ICM effectively alleviated these pathological changes and sepsis-induced cognitive impairment. Our study suggests that HMGB1 may be a target for the treatment of SAE.

## Data Availability

The datasets used and/or analysed during the current study are available from the corresponding author on reasonable request.

## Abbreviations

SAE: Sepsis-associated encephalopathy
HMGB1: High mobility group box-1 protein
CLP: Cecal ligation and puncture
ICM: Inflachromene
LTP: Long-term potentiation
CNS: Central nervous system
EEG: Electroencephalography
BBB: Blood‒brain barrier
AD: Alzheimer's disease
TBI: Traumatic brain injury
OFT: Open field test
NOR: Novel object recognition test
ACSF: Artificial cerebrospinal fluid
SC: Schaffer collateral
HFS: High-frequency stimulation
LPS: Lipopolysaccharide
